# Reproducibility of objectively measured physical activity and sedentary time over two seasons in children; Comparing a day-by-day and a week-by-week approach

**DOI:** 10.1371/journal.pone.0189304

**Published:** 2017-12-07

**Authors:** Eivind Aadland, Lars Bo Andersen, Turid Skrede, Ulf Ekelund, Sigmund Alfred Anderssen, Geir Kåre Resaland

**Affiliations:** 1 Faculty of Teacher Education and Sports, Campus Sogndal, Western Norway University of Applied Sciences, Norway; 2 Department of Sports Medicine, Norwegian School of Sport Sciences, Norway; 3 Center for Health Research, Førde Central Hospital, Norway; Universidad Europea de Madrid, SPAIN

## Abstract

**Introduction:**

Knowledge of reproducibility of accelerometer-determined physical activity (PA) and sedentary time (SED) estimates are a prerequisite to conduct high-quality epidemiological studies. Yet, estimates of reproducibility might differ depending on the approach used to analyze the data. The aim of the present study was to determine the reproducibility of objectively measured PA and SED in children by directly comparing a day-by-day and a week-by-week approach to data collected over two weeks during two different seasons 3–4 months apart.

**Methods:**

676 11-year-old children from the Active Smarter Kids study conducted in Sogn og Fjordane county, Norway, performed 7 days of accelerometer monitoring (ActiGraph GT3X+) during January-February and April-May 2015. Reproducibility was calculated using a day-by-day and a week-by-week approach applying mixed effect modelling and the Spearman Brown prophecy formula, and reported using intra-class correlation (ICC), Bland Altman plots and 95% limits of agreement (LoA).

**Results:**

Applying a week-by-week approach, no variables provided ICC estimates ≥ 0.70 for one week of measurement in any model (ICC = 0.29–0.66 not controlling for season; ICC = 0.49–0.67 when controlling for season). LoA for these models approximated a factor of 1.3–1.7 of the sample PA level standard deviations. Compared to the week-by-week approach, the day-by-day approach resulted in too optimistic reliability estimates (ICC = 0.62–0.77 not controlling for season; ICC = 0.64–0.77 when controlling for season).

**Conclusions:**

Reliability is lower when analyzed over different seasons and when using a week-by-week approach, than when applying a day-by-day approach and the Spearman Brown prophecy formula to estimate reliability over a short monitoring period. We suggest a day-by-day approach and the Spearman Brown prophecy formula to determine reliability be used with caution.

**Trial Registration:**

The study is registered in Clinicaltrials.gov 7^th^ April 2014 with identification number NCT02132494.

## Introduction

Objective assessment of movement has moved the field of physical activity (PA) monitoring substantially forward by replacing self-report measures suffering from many well-known limitations. Still, there are many unresolved issues regarding data reduction and quality assessment of data derived from accelerometry. This has resulted in great variation in procedures used and criteria applied to define what constitutes a valid measurement [1]. Behavior vary greatly over time. Thus, an important aspect of accelerometer measurements is how many days or periods of measurement are to be included to obtain reproducible estimates of habitual activity level. This is particularly true when children live in an area with a significant change in weather during different seasons [2–4]. As most diseases that can be prevented by PA develop over longer periods, the “true” habitual PA level would be more closely related to health than a short–for example a 7-day–snapshot. Association analyses will inherently suffer from severe regression dilution bias, if relying on a monitoring period that is too short [5]. Although the length of a period to be considered to constitute a person’s “habitual” or “regular” PA level is not easily defined, a 7-day period is arguably a short, and possible insufficient, period.

Most studies in children apply a criterion of a minimum 3 or 4 wear days to constitute a valid accelerometer-measurement period [1]. Although findings vary between studies in both adults [6–10] and children [11–22], most evidence suggest that a reasonable reliability (i.e., intra-class correlation (ICC)) of ~ 0.70–0.80 are achieved with 3–7 days of monitoring. Most previous studies have estimated the reliability of single days and thereafter calculated the number of days needed to reach a reasonable reliability level (often considered to be ICC = 0.80), based on the Spearman Brown prophecy formula for measurements conducted over a single 7-day period. Unfortunately, these study designs have received critique for being likely to underestimate the number of monitoring days needed, and their conclusions should therefore be interpreted with caution [23–25]. Importantly, these results are in principle only generalizable to the included days, as inclusion of additional days, weeks or seasons will add variability to the measurement and thus lower the reliability estimates for a given number of days (i.e., the variance partitioning of a fixed number of days to the total variance will decrease if the total variance increase).

Some studies have determined the reliability for several periods of measurement over the course of two weeks up to a year, of which all have shown considerable intra-individual variation [26, 27, 25, 28, 29]. Reliability has been shown to be ~ 0.70–0.80 for one out of two and three consecutive weeks of measurement in preschool children and adults, respectively [28, 29]. However, poorer estimates are found in studies including several seasons [26, 27, 25], leaving reliability estimates of ~ 0.50 for one week monitoring in children [27, 25]. Of particular interest, Wickel and Welk [25] showed that even applying three measurement periods across different seasons, did not result in a reliability of 0.80 using an absolute agreement definition (i.e., not controlling for season). This finding agrees with studies showing substantial seasonal variation in activity level in children and adolescents [2–4], which are obviously not captured when relying on a single measurement period. While the lower reliability estimates from these latter studies involving several monitoring periods might be due to variation across seasons, there might also be differences between the analytic approaches applied. To the best of our knowledge, no previous study has directly compared a day-by-day and a week-by-week approach for determining reliability for accelerometer outcomes, therefore we will address this important question. Furthermore, few studies have determined the intra-individual week-by-week reproducibility of accelerometer outcomes using absolute measures of agreement (i.e., limits of agreement (LoA) and/or standard error of the measurement (SEM)) [28, 29]. These previous studies should be extended to evaluation of agreement over different seasons.

The present study had two aims: 1) to determine the reproducibility of accelerometer-determined PA and sedentary time (SED) for one out of two 7-day measurement periods obtained during two different seasons separated by 3–4 months in a large sample of children; and 2) to directly compare a day-by-day and a week-by-week approach for analyzing reproducibility of accelerometer data. We hypothesized great variability across the monitoring periods for all accelerometer outcomes, resulting in reliability estimates lower than ICC = 0.80, and lower reliability using a week-by-week as compared to a day-by-day approach.

## Materials and methods

### Participants

The present analyses are based on data obtained in fifth grade children from the Active Smarter Kids (ASK) cluster-randomized trial, conducted in Norway during 2014–2015 [30, 31]. Physical activity was measured with accelerometry at baseline (mainly May to June 2014) and follow-up (April to May 2015) in all children, as well as in approximately two-thirds of the children that we invited to complete a mid-term measurement (January to February 2015). In the present study, we include the mid-term and the follow-up measurement, to allow for comparison of PA and SED over two different seasons separated by 3–4 months. Additionally, as the intervention was ongoing at both these time-points, we included both the intervention and the control groups. We have previously published a detailed description of the study [30], and do only provide a brief overview of the accelerometer handling herein.

Our procedures and methods conform to ethical guidelines defined by the World Medical Association’s Declaration of Helsinki and its subsequent revisions. The South-East Regional Committee for Medical Research Ethics approved the study protocol (reference number 2013/1893). We obtained written informed consent from each child’s parents or legal guardian and from the responsible school authorities prior to all testing. The study is registered in Clinicaltrials.gov with identification number: NCT02132494.

### Procedures

Physical activity was measured using the ActiGraph GT3X+ accelerometer (Pensacola, FL, USA) [32]. During both measurements, participants were instructed to wear the accelerometer at all times over 7 consecutive days, except during water activities (swimming, showering) or while sleeping. Units were initialized at a sampling rate of 30 Hz. Files were analyzed at 10 second epochs using the KineSoft analytical software version 3.3.80 (KineSoft, Loughborough, UK). Data was restricted to hours 06:00 to 23:59. In all analyses, consecutive periods of ≥ 20 minutes of zero counts were defined as non-wear time [33, 1]. Results are reported for overall PA level (cpm), as well as minutes per day spent SED (< 100 cpm), in light PA (LPA) (100–2295 cpm), in moderate PA (MPA) (2296–4011 cpm), in vigorous PA (VPA) (≥ 4012 cpm), and in moderate-to-vigorous PA (MVPA) (≥ 2296 cpm), determined using previously established and validated cut points [34, 35]. We reported main results for four different wear time requirements (≥ 8 and ≥ 10 hours/day, and ≥ 3 and ≥ 5 days/week), and included sensitivity analyses requiring the inclusion of both weekdays and weekend days (≥ 3 weekdays and ≥ 1 weekend day, and ≥ 4 weekdays and 2 weekend days).

### Statistical analyses

Children’s characteristics were reported as frequencies, means and standard deviations (SD). Differences between included and excluded children, differences in PA level between measurements, and differences in intra-individual variation for the combined period (14 days) against the mean of the two separate weeks was tested using a mixed effect model including random intercepts for children. Wear time was included as a covariate for analyses of PA and SED.

We estimated reliability using two approaches; 1) day-by-day analyses, and 2) week-by-week analyses. In both approaches, reliability for single days (day-by-day approach) and weeks (week-by-week approach) of measurement (ICC_s_) was assessed using variance partitioning applying a one-way random effect model not controlling for season (i.e., determining reliability based on an absolute agreement definition) and a two-way mixed effect model controlling for season (i.e., determining reliability based on a consistency definition) [36]. All models were adjusted for wear time by adding wear time as a covariate, as wear time has a strong association with PA and SED estimates and also impact reliability [29], and since most studies control for wear time. The number of days (day-by-day approach) and weeks (week-by-week approach) needed to obtain a reliability of 0.80 (N) was estimated using the Spearman Brown prophecy formula (ICC for average measurements [ICC_k_]) [6, 36]: N = ICC_t_/(1-ICC_t_)*[(1-ICC_s_)/ICC_s_], where N = the number of days or weeks needed, ICC_t_ = the desired level of reliability, and ICC_s_ = the reliability for single days or weeks. Additionally, the ICC_k_ (between-subject variance/[between-subject variance + residual variance/k]) for k = 6 (i.e., the mean number of monitoring days/week) was calculated to directly compare reliability estimates for one week of measurement from the day-by-day and the week-by-week approach.

In the week-by-week analyses, we additionally applied Bland Altman plots, showing the difference between two subsequent weeks as a function of the mean of the two weeks [37], to visualize the week-by-week measurement variability. We calculated 95% LoA and coefficient of variation (CV) from the residual variance (i.e., within-subjects) error term based on the variance partitioning models (LoA = √residual variance *√2*1.96; CV = √residual variance/mean values) [38]. We assessed whether the variability varied as a function of the mean activity levels (i.e., whether data were homoscedastic or heteroscedastic) by correlating absolute differences against the mean values using Pearson’s correlation coefficient (r). For quantification of measurement error, an absolute measure of error (e.g., LoA) provide the correct estimate for homoscedastic data (where there are no association between variability and mean values), whereas a relative measure of error (e.g., CV) provide the correct estimate for heteroscedastic data (where variability increases with increased mean values) [39]. Yet, both measures provide valid reliability estimates for the mean sample PA levels.

All analyses were performed using IBM SPSS v. 23 (IBM SPSS Statistics for Windows, Armonk, NY: IBM Corp., USA). A p-value < .05 indicated statistically significant findings.

## Results

### Participants’ characteristics

Of the 1129 children included in the ASK-study, 676 children provided accelerometer data at the mid-term and post measurement, of whom 615 children (50% boys) fulfilled the ≥ 480 minutes/day and ≥ 3 days/week wear criterion (Table 1). There were no differences between the included (n = 615) and excluded (n = 514) children in anthropometry (p ≥ .092) or PA level at the post measurement (p ≥ .218). For the included children, the number of wear days was similar between the winter and spring measurement, whereas the valid wear time was marginally higher during the spring measurement. Overall PA level (cpm) and intensity-specific PA was significantly higher (except for LPA in girls), and SED was significantly lower, in the spring than in the winter for both boys and girls. The greatest increase from the winter to the spring measurement was seen for VPA (50% in boys and 44% in girls), overall PA level (31% in boys and 26% in girls), and MVPA (28% in boys and 23% in girls).

**Table 1:** The children’s characteristics. Values are mean (SD) if not otherwise stated.

	Boys (n = 309)			Girls (n = 306)			Total (n = 615)
**Age (years)**	10.9 (0.3)			10.9 (0.3)			10.9 (0.3)
**Body mass (kg)**	39.0 (8.4)			39.7 (9.1)			39.4 (8.8)
**Height (cm)**	146 (7)			146 (7)			146 (7)
**BMI (kg/m**^**2**^**)**	18.1 (2.9)			18.4 (3.2)			18.2 (3.1)
**Overweight/obese (%)**	17/4			15/4			16/4
Physical activity level*	**Winter**	**Spring**	P**	**Winter**	**Spring**	P**	**Combined**
**Wear days (days/week)**	6.0 (1.1)	6.0 (1.2)	.624	6.1 (1.1)	6.1 (1.1)	.559	6.0 (1.2)
**Wear minutes (min/day)**	771 (55)	780 (55)	.010	773 (47)	781 (52)	.015	776 (53)
**Overall PA (cpm)**	510 (131)	666 (206)	< .001	456 (116)	575 (176)	< .001	552 (184)
**SED (min/day)**	499 (56)	485 (56)	< .001	508 (47)	501 (55)	< .001	498 (54)
**LPA (min/day)**	210 (35)	217 (34)	.002	215 (32)	219 (33)	.164	216 (34)
**MPA (min/day)**	38 (11)	44 (12)	< .001	31 (8)	35 (10)	< .001	37 (12)
**VPA (min/day)**	20 (10)	30 (14)	< .001	16 (9)	23 (12)	< .001	22 (13)
**MVPA (min/day)**	58 (19)	74 (24)	< .001	47 (15)	58 (21)	< .001	59 (22)

BMI = body mass index; PA = physical activity; cpm = counts per minute; SED = sedentary time; LPA = light physical activity; MPA = moderate physical activity; VPA = vigorous physical activity; MVPA = moderate-to-vigorous physical activity

* All results are based on weekly means using a ≥ 480 minutes/day & ≥ 3 days/week criterion. Total n = 615; 308 boys and 306 girls; n = 592–615 for variables other than physical activity as some children had missing data.

**P-values for winter vs. spring.

### Reliability based on a day-by-day approach

Table 2 shows the reliability for single days of measurement (ICC_s_) and the number of days (N) needed to achieve a reliability of 0.80, as estimated by the Spearman Brown prophecy formula. For all variables, reliability increased marginally (N decreased by 0.1–0.8 days) when applying a stricter wear time criteria (10 hours/day vs. 8 hours/day). For intensity-specific PA and SED, reliability was marginally better during the winter (N was 0.1–2.4 days lower than in the spring), whereas a profound difference was found for overall PA level (cpm), for which N ~ 7 days at winter and ~ 12 days at spring (S1 Table). The mean intra-individual SDs increased by 4.2–13.9% across variables when including two weeks of measurement compared to the mean of the two separate weeks (Overall PA: 221 vs. 194 cpm, p < .001; SED: 79.3 vs. 76.1 min/day, p < .001; LPA: 42.5 vs. 40.7 min/day, p < .001; MPA: 14.9 vs. 14.0 min/day, p < .001; VPA: 14.0 vs 12.5 min/day, p < .001; MVPA: 26.8 vs 24.7 min/day, p < .001). Consistent with this increased variation, reliability estimates decreased when analyzing the overall 14-day period compared to either of the two weeks. When applying the whole 14-day period, we estimated that 7–15 and 7–14 days of measurement was needed to reach a reliability level of 0.80 when not controlling for season and controlling for season, respectively.

**Table 2:** Reliability for single days of measurement (ICC_s_) and number of days needed to achieve a reliability of 0.80 (N) for the two weeks (winter and spring).

	1 week, mean estimates		2 weeks, not adjusted for season		2 weeks, adjusted for season	
	ICC_s_	N	ICC_s_	N	ICC_s_	N
	**≥ 8 hours/day wear criterion (n = 615 children, 7441 days)**					
**Overall PA (cpm)**	0.29	10.1	0.21	14.8	0.23	13.6
**SED (min/day)**	0.35	7.3	0.32	8.5	0.33	8.3
**LPA (min/day)**	0.40	6.0	0.36	7.0	0.36	7.0
**MPA (min/day)**	0.32	8.5	0.28	10.1	0.29	10.0
**VPA (min/day)**	0.34	7.8	0.28	10.2	0.30	9.5
**MVPA (min/day)**	0.34	7.9	0.30	9.5	0.31	9.0
	**≥ 10 hours/day wear criterion (n = 587 children, 6745 days)**					
**Overall PA (cpm)**	0.30	9.5	0.22	13.9	0.24	12.8
**SED (min/day)**	0.36	7.0	0.33	8.1	0.33	8.0
**LPA (min/day)**	0.41	5.7	0.37	6.8	0.37	6.8
**MPA (min/day)**	0.33	8.1	0.29	9.8	0.29	9.6
**VPA (min/day)**	0.35	7.5	0.29	10.0	0.30	9.2
**MVPA (min/day)**	0.34	7.6	0.30	9.2	0.31	8.7

PA = physical activity; cpm = counts per minute; SED = sedentary time; LPA = light physical activity; MPA = moderate physical activity; VPA = vigorous physical activity; MVPA = moderate-to-vigorous physical activity; ICC_s_ = intra-class correlation for a single day of measurement adjusted for wear time; N = number of days needed to achieve an ICC = 0.80; all estimates are based on a ≥ 3 days wear time criterion.

### Reliability based on a week-by-week approach

**Fig 1:**
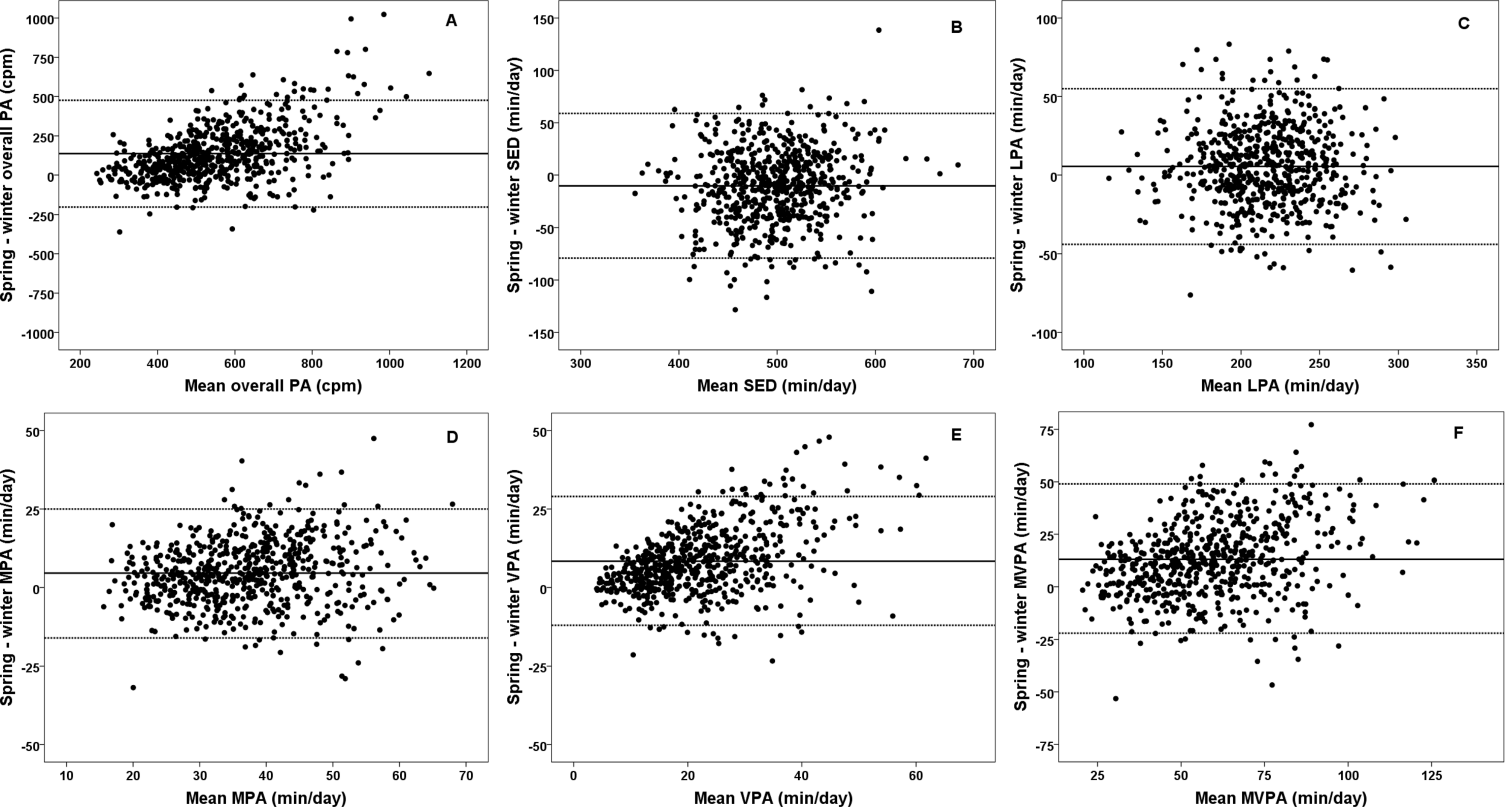
Bland Altman plots of agreement for different outcome variables over two weeks of measurement performed in the winter and spring, 3 to 4 months apart. Bland Altman plots (the mean of two weeks of measurement on the x-axis versus the difference between them on the y-axis) for (a) overall physical activity (cpm), and minutes per day spent (b) sedentary (SED), (c) in light physical activity (LPA), (d) in moderate physical activity (MPA), (e) in vigorous physical activity (VPA), and (f) in moderate-to-vigorous physical activity (MVPA). Results are based on a ≥ 8 hours & ≥ 3 days wear time criterion (n = 615). The full line is the bias between weeks, whereas the dotted lines are 95% limits of agreement corrected for wear time and season.

We found minor improvements in week-by-week reliability when data was accumulated over longer daily wear time (≥ 8 to ≥ 10 hours) and more days (≥ 3 to ≥ 5 days) (Table 3), and when requiring both week and weekend days (S2 Table). The bias (spring—winter) between the weeks was in average 137 (95% CI; 124–151) (p < .001) cpm for overall PA, and -10.2 (-14.3–-6.1) (p < .001), 5.5 (3.2–7.8) (p < .001), 4.6 (3.7–5.4) (p < .001), 8.4 (7.6–9.3) (p < .001), and 13.1 (11.6–14.5) (p < .001) min/day for SED, LPA, MPA, VPA, and MVPA, respectively. As shown in Table 3, no variables provided ICC estimates ≥ 0.70 for one week of measurement in any model, values being 0.29–0.66 when not controlling for season (using an absolute agreement definition), and 0.49–0.67 when controlling for season (using a consistency definition), indicating substantial intra-individual variation over time for all outcomes, as shown in Fig 1. Agreement (LoA) for these models approximated a factor of 1.3–1.7 the sample PA level SDs. CVs were small to moderate for SED (0.05–0.06) and LPA (0.08–0.09), but large for MPA (0.19–0.22), VPA (0.33–0.44), MVPA (0.21–0.27), as well as overall PA (0.21–0.28). Variability increased with increased activity level for overall PA level (r for absolute differences vs. mean activity level = 0.56, p < .001), MPA (r = 0.27, p< .001), VPA (r = 0.55, p < .001) and MVPA (r = 0.39, p < .001), but not for SED and LPA (r = -0.05–0.06, p ≥.152). The number of weeks needed to reach a reliability level of 0.80 was 2–10 when not controlling for season, and 2–4 when controlling for season. Overall PA level was clearly the least reliable outcome across models.

**Table 3:** The week-by-week reproducibility for different outcome variables for two weeks of measurement.

	≥ 8 hours/day								≥ 10 hours/day							
	≥ 3 days/week (n = 615 (91%) children)				≥ 5 days/week (n = 482 (71%) children)				≥ 3 days/week (n = 587 (87%) children)				≥ 5 days/week (n = 420 (62%) children)			
	ICC_s_	LoA	N	CV	ICC_s_	LoA	N	CV	ICC_s_	LoA	N	CV	ICC_s_	LoA	N	CV
**Not corrected for season (absolute agreement definition)**																
**Overall PA (cpm)**	0.29	430.9	9.9	0.28	0.32	415.7	8.7	0.27	0.29	425.6	9.6	0.28	0.32	407.4	8.5	0.27
**SED (min/day)**	0.59	75.6	2.7	0.05	0.62	72.3	2.4	0.05	0.60	77.2	2.7	0.06	0.62	74.6	2.5	0.05
**LPA (min/day)**	0.65	50.1	2.2	0.08	0.66	48.9	2.0	0.08	0.65	51.1	2.1	0.09	0.66	50.1	2.0	0.08
**MPA (min/day)**	0.53	21.8	3.5	0.21	0.56	20.4	3.1	0.20	0.53	22.5	3.6	0.22	0.55	21.2	3.3	0.21
**VPA (min/day)**	0.42	26.1	5.4	0.43	0.43	25.7	5.2	0.42	0.42	26.9	5.5	0.44	0.44	25.8	5.1	0.42
**MVPA (min/day)**	0.50	42.9	4.0	0.26	0.53	41.0	3.6	0.25	0.50	44.1	4.0	0.27	0.53	41.9	3.6	0.26
**Corrected for season (consistency definition)**																
**Overall PA (cpm)**	0.49	338.7	4.2	0.22	0.51	326.6	3.8	0.21	0.48	339.0	4.3	0.22	0.52	320.4	3.8	0.21
**SED (min/day)**	0.65	69.2	2.2	0.05	0.67	66.9	2.0	0.05	0.65	71.0	2.2	0.05	0.66	69.2	2.1	0.05
**LPA (min/day)**	0.65	49.8	2.1	0.08	0.66	48.7	2.0	0.08	0.66	50.8	2.1	0.08	0.67	50.0	2.0	0.08
**MPA (min/day)**	0.58	20.2	2.9	0.20	0.60	19.2	2.7	0.19	0.58	20.9	3.0	0.20	0.59	19.8	2.7	0.19
**VPA (min/day)**	0.59	20.7	2.7	0.34	0.61	20.2	2.6	0.33	0.58	21.6	2.8	0.35	0.61	20.3	2.5	0.33
**MVPA (min/day)**	0.63	35.4	2.3	0.22	0.65	33.9	2.1	0.21	0.62	36.8	2.4	0.23	0.65	34.7	2.2	0.21

PA = physical activity; cpm = counts per minute; SED = sedentary time; LPA = light physical activity; MPA = moderate physical activity; VPA = vigorous physical activity; MVPA = moderate-to-vigorous physical activity; ICC_s_ = intra-class correlation for a single week of measurement adjusted for wear time; N = number of weeks needed to achieve a ICC = 0.80; LoA = 95% limits of agreement; CV = coefficient of variation

Reliability was similar for the intervention and control groups, the maximum difference being ICC = 0.05 across outcomes and models.

### Comparison of reliability estimates across approaches

As reliability estimates differed between the day-by-day and the week-by-week approaches, we show a direct comparison of estimates for these approaches in Table 4. Estimates using the day-by-day approach are averaged over 6 monitoring days, thus being similar to the weekly averages in terms of the number of monitoring days included. Despite both calculations were based on the exact same data, reliability estimates was substantially higher using the day-by-day approach (ICC = 0.62–0.77), compared to the week-by-week approach (ICC = 0.29–0.65).

**Table 4:** The week-by-week reliability for different outcome variables for two weeks of measurement.

	Day-by-day approach			Week-by-week approach		
	Variance components		ICC_k6_	Variance components		ICC_s_
	Subject	Residual_k6_		Subject	Residual	
	**Not corrected for season (absolute agreement definition)**					
**Overall PA (cpm)**	17027	10535	0.62	9737	24170	0.29
**SED (min/day)**	1239	438	0.74	1088	744	0.59
**LPA (min/day)**	666	195	0.77	602	327	0.65
**MPA (min/day)**	83.4	35.2	0.70	70.1	62.1	0.53
**VPA (min/day)**	92.6	39.5	0.70	65.5	88.7	0.42
**MVPA (min/day)**	305	121	0.72	242	240	0.50
	**Corrected for season (consistency definition)**					
**Overall PA (cpm)**	17130	9710	0.64	14312	14933	0.49
**SED (min/day)**	1241	429	0.74	1149	623	0.65
**LPA (min/day)**	666	195	0.77	604	322	0.65
**MPA (min/day)**	83.3	34.6	0.71	74.3	53.3	0.58
**VPA (min/day)**	92.5	36.5	0.72	81.8	55.7	0.59
**MVPA (min/day)**	305	114	0.73	280	163	0.63

PA = physical activity; cpm = counts per minute; SED = sedentary time; LPA = light physical activity; MPA = moderate physical activity; VPA = vigorous physical activity; MVPA = moderate-to-vigorous physical activity; _k6_ = estimates are calculated based on an ICC for average measurements over 6 monitoring days, which was the mean number of valid days for one week of monitoring in the present study; ICC_s_ = intra-class correlation for a single week of measurement; all estimates are based on a ≥ 8 hours & ≥ 3 days wear time criterion, and are adjusted for wear time

## Discussion

The present study aimed to determine the reproducibility of accelerometer-determined PA and SED over two different seasons and to directly compare a day-by-day and a week-by-week approach for analyzing reproducibility of accelerometer data. Our results suggest that 1) the reliability for one out of two week-long measurements undertaken 3–4 months apart resulted in estimates clearly lower than most previous studies that have relied on a single monitoring period, and that 2) a day-by-day approach overestimated the reliability compared to a week-by-week approach. Our findings indicate that the children’s PA level varied up to ± 1.3–1.7 SD units between the two measurements, indicating substantial measurement error for all variables.

Most previous studies investigating reliability and the required number of accelerometer monitoring days have estimated reliability based on day-by-day analyses using a single 7-day monitoring period [11, 16, 17, 40, 18, 19, 22, 20, 21, 12–15]. In general, these studies conclude that 3–7 monitoring days are sufficient in children. This approach, however, restricts variation and underestimates the number of monitoring days and periods needed to obtain reliable estimates. We applied two monitoring periods covering two different seasons, leading to findings very similar to previous studies that have applied multiple measurement periods over the course of several seasons. These studies have yielded substantially lower reliability estimates in adults [26] and children [27, 25], concluding that more than one monitoring period is needed to reach a reliability level of 0.80. Mattocks [27] determined overall PA, MVPA and SED over four 7-day periods over approximately one year using the Actigraph 7164 accelerometer in 11–12-year-old children. The ICC for one period of measurement varied from 0.45 to 0.59 across outcome variables. Wickel & Welk [25] found an ICC of 0.46 for one out of three 7-day periods to assess steps for the Digiwalker pedometer in 80 children aged ~ 10 years. The present findings along with these previous findings question the validity of one week of measurement to determine children’s “true” habitual activity level.

Whereas we found that 7–15 days of measurement was required to reach a reliability of 0.80 based on the day-by-day analyses, 2–10 weeks of measurement was required based on the week-by-week analyses. These contrasting findings strengthen the argument that the estimation of number of days needed using the traditional approach, that is, applying the Spearman Brown prophecy formula to single days, might be used with caution. We have no explanation for these contrasting findings, but our findings do support previous studies that have warned against a possible overestimation of reliability by the day-by-day approach [23–25]. This is especially clear when the assessment is spread across different seasons. For example, two studies have revealed similar results for a day-to-day and a week-to-week approach [28, 29]. However, contrary to the present study, these studies were based on two consecutive weeks of measurement. In contrast, both the present study and others that have introduced multiple seasons [27, 25], found increased variability in estimates. Apparently, seasonality has a more profound effect on the week-by-week analysis than the day-by-day analysis, as illustrated by the differences in reliability estimates with and without controlling for season shown in Table 4. The difference in variance between the two monitoring periods (Table 1) could explain the findings, as the model assumes compound symmetry and the ICC are sensitive to asymmetry [36], however, this difference between measurements applies to both analytic approaches. Nevertheless, it is clear that applying the Spearman Brown prophecy formula/the ICC_k_ calculated for average days [36], which imply dividing the residual variance over the desired number of days, seems overly optimistic when compared to week-by-week approach. Notably, this limitation also applies to the estimation of the number of weeks needed for the week-by-week approach.

As noise in exposure (x) variables will lead to attenuation of regression coefficients (regression dilution bias), and noise in outcome (y) variables will increase standard errors [5], unreliable measures weaken researchers ability to make valid conclusions. In epidemiology, researchers are in general interested in the long-term “true” habitual PA level, rather than activity during the most recent days. There are some health characteristics, as for example insulin resistance, lipid metabolism and blood pressure, that might change with acute increases or decreases in PA [41]. Despite this, a child’s level of fatness, aerobic fitness or motor skill takes months or years to change. For such stable traits, association analyses will inherently suffer from regression dilution bias if relying on a 7-day monitoring period that provide an insufficient snapshot of children’s habitual activity level. Similarly, tracking coefficients for PA are generally low to moderate [42–44], probably due to measurement error as much as true change over time. Interestingly, our reliability estimates over 3–4 months are quite similar to many tracking estimates reported in the literature. This finding challenge our understanding of behavioral change versus measurement error, as they are both different sides of the same coin.

Although an increased monitoring length might improve validity of study conclusions, the burden for participants should be kept minimal to maximize response rate and compliance. We have previously performed 2 and 3-week monitoring protocols in preschool children and adults, respectively, without any major issues regarding compliance [28, 29]. More recently, we have also successfully performed a 2-week monitoring protocol in larger samples of children, adults and older people, demonstrating this protocol’s acceptance in various context. Still, performing measurements over separate as opposed to consecutive periods might pose an increased burden for participants, as well as for researchers. Notably, the required monitoring volume is a matter of the research question posed, as population-estimates on a group level requires a lower level of reproducibility than individual-level estimates used for association analyses [24].

### Strengths and limitations

The main strength of the present study is the inclusion of a large and representative sample of children. As reliability estimates (i.e., ICCs) depend on the sample variation [37, 45, 38], the validity of the estimated ICCs presented herein should be generalizable to other contexts, including large-scale population studies. Another strength is inclusion of measurements conducted 3–4 months apart, during two different seasons. Thus, these data clearly serve the aim of the study; we introduced more variability than within a shorter time frame, but also restricting the duration to some few months, where “true” changes over time would be expected to be limited. A limitation, though, is the inclusion of only two weeks and two seasons, as inclusion of more observations probably would introduce more variability and lead to more conservative reproducibility estimates [27, 25]. Moreover, Norway has profound seasonal differences in weather conditions. This characteristic might limit generalizability to areas with less pronounced seasonality. Finally, the inclusion of the intervention group in the current analyses might have caused additional variation to the data, as the intervention group could be expected to change their PA level over time. Yet, the intervention was ongoing during both measurements, there was no effect of the intervention on PA levels [31], and reliability estimates differed marginally between the intervention and control groups.

## Conclusion

We conclude that a one-week accelerometer monitoring period conducted during two different seasons 3–4 months apart resulted in modest reproducibility between measurements in a large sample of children (ICC for one week = 0.32–0.67). The traditional approach for estimating the number of wear days needed for accelerometer measurements–applying the Spearman Brown prophecy formula to single days of measurement over a short monitoring period–resulted in more optimistic reliability estimates than a week-by-week approach. Thus, consistent with previous studies that have raised concern about the traditional approach to estimate reliability of accelerometer monitoring protocols, we suggest results from studies using a day-by-day approach to determine reliability be interpreted with caution. Researchers should consider increasing the monitoring period beyond a single 7-day period in future studies.

## Supporting information

S1 File The data file underlying the study findings. (XLSX)

S1 Table Reliability for single days of measurement (ICC_s_) and number of days needed to achieve a reliability of 0.80 (N) for the two weeks (winter and spring) separately. (DOCX)

S2 Table The week-by-week reliability for one out of two weeks of measurement for different wear criteria requiring both weekdays (3 or 4 days) and weekend days (1 or 2 days). (DOCX)
