# Supplementary material for: Reproducibility of objectively measured physical activity and sedentary time over two seasons in children; Comparing a day-by-day and a week-by-week approach
Source: PLoS One. 2017 Dec 7;12(12):e0189304. doi: 10.1371/journal.pone.0189304 (PMC5720738; doi:10.1371/journal.pone.0189304)
Supplement: S1 Table — (DOCX) [file pone.0189304.s002.docx]

**S1 Table. Reliability for single days of measurement (ICC_s_) and number of days needed to achieve a reliability of 0.80 (N) for the two weeks (winter and spring) separately***.*

|  | **Winter (7 days)** | | **Spring (7 days)** | |
| --- | --- | --- | --- | --- |
|  | **ICC_s_** | **N** | **ICC_s_** | **N** |
|  | **≥ 8 hours/day wear criterion (n = 615 children, 7441 days)** | | | |
| **Overall PA (cpm)** | 0.33 | 8.0 | 0.25 | 12.1 |
| **SED (min/day)** | 0.37 | 6.9 | 0.34 | 7.8 |
| **LPA (min/day)** | 0.41 | 5.7 | 0.39 | 6.2 |
| **MPA (min/day)** | 0.32 | 8.4 | 0.32 | 8.4 |
| **VPA (min/day)** | 0.36 | 7.1 | 0.32 | 8.5 |
| **MVPA (min/day)** | 0.34 | 7.6 | 0.33 | 8.2 |
|  | **≥ 10 hours/day wear criterion (n = 587 children, 6745 days)** | | | |
| **Overall PA (cpm)** | 0.35 | 7.5 | 0.26 | 11.5 |
| **SED (min/day)** | 0.38 | 6.6 | 0.35 | 7.4 |
| **LPA (min/day)** | 0.42 | 5.6 | 0.40 | 5.9 |
| **MPA (min/day)** | 0.33 | 8.0 | 0.33 | 8.3 |
| **VPA (min/day)** | 0.37 | 6.7 | 0.32 | 8.4 |
| **MVPA (min/day)** | 0.36 | 7.2 | 0.33 | 8.0 |

PA = physical activity; cpm = counts per minute; SED = sedentary time; LPA = light physical activity; MPA = moderate physical activity; VPA = vigorous physical activity; MVPA = moderate-to-vigorous physical activity; ICC_s_ = intra-class correlation for a single day of measurement adjusted for wear time; N = number of days needed to achieve a ICC = 0.80
