# Supplementary material for: Reproducibility of objectively measured physical activity and sedentary time over two seasons in children; Comparing a day-by-day and a week-by-week approach
Source: PLoS One. 2017 Dec 7;12(12):e0189304. doi: 10.1371/journal.pone.0189304 (PMC5720738; doi:10.1371/journal.pone.0189304)
Supplement: S2 Table — (DOCX) [file pone.0189304.s003.docx]

**S2 Table. The week-by-week reliability for one out of two weeks of measurement for different wear criteria requiring both weekdays (3 or 4 days) and weekend days (1 or 2 days).**

|  | **≥ 8 hours/day** | | | | | | | | **≥ 10 hours/day** | | | | | | | |
| --- | --- | --- | --- | --- | --- | --- | --- | --- | --- | --- | --- | --- | --- | --- | --- | --- |
|  | **≥ 3 days/week**  **(n = 496 (73%) children)** | | | | **≥ 5 days/week**  **(n = 294 (44%) children)** | | | | **≥ 3 days/week**  **(n = 426 (63%) children)** | | | | **≥ 5 days/week**  **(n = 178 (26%) children)** | | | |
|  | **ICC_s_** | **LoA** | **N** | **CV** | **ICC_s_** | **LoA** | **N** | **CV** | **ICC_s_** | **LoA** | **N** | **CV** | **ICC_s_** | **LoA** | **N** | **CV** |
| **Not corrected for season (absolute agreement definition)** | | | | | | | | | | | | | | | | |
| **Overall PA (cpm)** | 0.31 | 420 | 8.8 | 0.27 | 0.30 | 412 | 9.3 | 0.27 | 0.30 | 419 | 9.3 | 0.27 | 0.35 | 392 | 7.5 | 0.26 |
| **SED (min/day)** | 0.61 | 73.5 | 2.5 | 0.05 | 0.65 | 70.0 | 2.2 | 0.05 | 0.60 | 76.3 | 2.7 | 0.06 | 0.67 | 67.6 | 2.0 | 0.05 |
| **LPA (min/day)** | 0.66 | 48.9 | 2.0 | 0.08 | 0.69 | 48.8 | 1.8 | 0.08 | 0.65 | 51.0 | 2.1 | 0.09 | 0.70 | 48.2 | 1.7 | 0.08 |
| **MPA (min/day)** | 0.55 | 21.0 | 3.2 | 0.20 | 0.59 | 19.1 | 2.8 | 0.19 | 0.55 | 21.4 | 3.2 | 0.21 | 0.60 | 19.3 | 2.7 | 0.19 |
| **VPA (min/day)** | 0.44 | 25.8 | 5.0 | 0.42 | 0.43 | 24.9 | 5.2 | 0.41 | 0.43 | 26.5 | 5.3 | 0.43 | 0.45 | 25.3 | 4.9 | 0.41 |
| **MVPA (min/day)** | 0.53 | 41.7 | 3.6 | 0.25 | 0.55 | 38.8 | 3.3 | 0.24 | 0.52 | 43.0 | 3.8 | 0.26 | 0.56 | 39.6 | 3.1 | 0.24 |
| **Corrected for season (consistency definition)** | | | | | | | | | | | | | | | | |
| **Overall PA (cpm)** | 0.52 | 327 | 3.7 | 0.21 | 0.51 | 318 | 3.8 | 0.21 | 0.52 | 320 | 3.7 | 0.21 | 0.55 | 302 | 3.3 | 0.20 |
| **SED (min/day)** | 0.67 | 67.3 | 2.0 | 0.05 | 0.69 | 65.2 | 1.8 | 0.05 | 0.66 | 69.0 | 2.1 | 0.05 | 0.71 | 63.2 | 1.7 | 0.05 |
| **LPA (min/day)** | 0.67 | 48.6 | 2.0 | 0.08 | 0.69 | 48.8 | 1.8 | 0.08 | 0.66 | 50.6 | 2.1 | 0.08 | 0.70 | 48.3 | 1.8 | 0.08 |
| **MPA (min/day)** | 0.60 | 19.5 | 2.7 | 0.19 | 0.63 | 17.9 | 2.3 | 0.17 | 0.61 | 19.5 | 2.5 | 0.19 | 0.65 | 17.8 | 2.1 | 0.17 |
| **VPA (min/day)** | 0.61 | 20.2 | 2.5 | 0.33 | 0.62 | 19.2 | 2.5 | 0.31 | 0.62 | 20.3 | 2.5 | 0.33 | 0.63 | 19.5 | 2.3 | 0.32 |
| **MVPA (min/day)** | 0.65 | 34.3 | 2.1 | 0.21 | 0.68 | 31.3 | 1.9 | 0.19 | 0.66 | 34.3 | 2.1 | 0.21 | 0.70 | 31.5 | 1.7 | 0.19 |

PA = physical activity; cpm = counts per minute; SED = sedentary time; LPA = light physical activity; MPA = moderate physical activity; VPA = vigorous physical activity; MVPA = moderate-to-vigorous physical activity; ICC_s_ = intra-class correlation for a single week of measurement adjusted for wear time; N = number of weeks needed to achieve a ICC = 0.80; LoA = 95% limits of agreement; CV = coefficient of variation
